# Contagion and Chaos: Disease, Ecology, and National Security in the Era of Globalization

**DOI:** 10.3201/eid1511.090577

**Published:** 2009-11

**Authors:** Stephen A. Morse

**Affiliations:** Centers for Disease Control and Prevention, Atlanta, Georgia, USA

**Keywords:** Epidemics, SARS, pandemic influenza, influenza, bioterrorism and preparedness, book review

Contagion and Chaos describes the threat that emerging and reemerging infectious diseases pose to international security because of these diseases’ negative effects on sovereign states. The author proposes the following 5 hypotheses: 1) epidemic disease may compromise the prosperity, legitimacy, structural cohesion, and, in certain cases, security of sovereign states; 2) epidemics and pandemics of emerging or reemerging infectious diseases may promote economic and political discord among countries but are unlikely to generate serious armed conflict; 3) only some pathogens threaten national security according to criteria such as lethality, transmissibility, fear, and economic damage; 4) warfare (intrastate and interstate) amplifies problems caused by disease; and 5) the paradigm of health security is philosophically grounded in the political tradition of republican theory.

The author stresses that the association between the health of a population and perception of national security is ancient but largely forgotten. He suggests that a republican revision of systems-level international relations theory provides an optimal framework for examining the paradigm of health security.

The book’s 8 chapters discuss data supporting the author’s hypotheses. The first chapter describes the relationships among pathogens, society, and the state from a political science perspective. For nonpolitical scientists, this chapter is difficult. However, chapters 2–7 are interesting and enlightening. Chapter 2 explores the historical relationship between the state and society in the context of contagion. The author provides a historical perspective for the long-held perception that infectious disease poses a distinct threat to the stability, prosperity, material interests, and, therefore, security of the state. Chapters 3–6 present case studies concerning the influenza pandemic of 1918, HIV/AIDS, bovine spongiform encephalopathy and its human variant, Creutzfeld-Jakob disease, and severe acute respiratory syndrome. Each illness is discussed in the context of disease-induced destruction and debilitation of the population, erosion of productivity and prosperity, fear-induced social destabilization, disruption of governance institutions, and the consequent erosion of state power relative to unaffected rival states. In Chapter 7, violent conflict and war are shown to be disease amplifiers through examination of the mechanisms by which interstate and intrastate conflict contributes to disseminating existing pathogens and to emerging novel microorganisms. The final chapter examines the proposition that health contributes to economic prosperity, which bolsters the power of the state. Each chapter has extensive notes to assist the reader.

The author proposes that the best way to curtail future epidemics (and pandemics) is to augment the healthcare infrastructure and improve the health of populations. Fulfilling these needs is particularly important for developing countries where conditions are favorable for disease emergence because of globalization that results in increased population density, ecological degradation, rapid transportation technologies, and mass migration and because of low or nonexistent disease surveillance and containment capacities. This book will be of interest to political scientists and those in public health and medicine because it highlights the interdependence between political science and public health.

